# Generation of immunocompetent somatic glioblastoma mouse models through *in situ* transformation of subventricular zone neural stem cells

**DOI:** 10.1016/j.xpro.2024.102928

**Published:** 2024-03-01

**Authors:** Melanie Clements, Holly Simpson Ragdale, Claudia Garcia-Diaz, Simona Parrinello

**Affiliations:** 1 Samantha Dickson Brain Cancer Unit, UCL Cancer Institute, WC1E 6DD London, UK

**Keywords:** Cancer, Model Organisms, CRISPR, Stem Cells

## Abstract

Disease-relevant *in vivo* tumor models are essential tools for both discovery and translational research. Here, we describe a highly genetically tractable technique for generating immunocompetent somatic glioblastoma (GBM) mouse models using piggyBac transposition and CRISPR-Cas9-mediated gene editing in wild-type mice. We describe steps to deliver plasmids into subventricular zone endogenous neural stem cells by injection and electroporation, leading to the development of adult tumors that closely recapitulate the histopathological, molecular, and cellular features of human GBM.

For complete details on the use and execution of this protocol, please refer to Garcia-Diaz et al.^1^

## Highlights

• Protocol for generation of somatic glioblastoma mouse models in wild-type mice
• piggyBac technology for delivery of human driver mutations to SVZ neural stem cells
• Procedure for intraventricular injection and electroporation of piggyBac plasmids

## Abstract (editor highlights)

Publisher’s note: Undertaking any experimental protocol requires adherence to local institutional guidelines for laboratory safety and ethics.

## Graphical abstract

**Figure:**
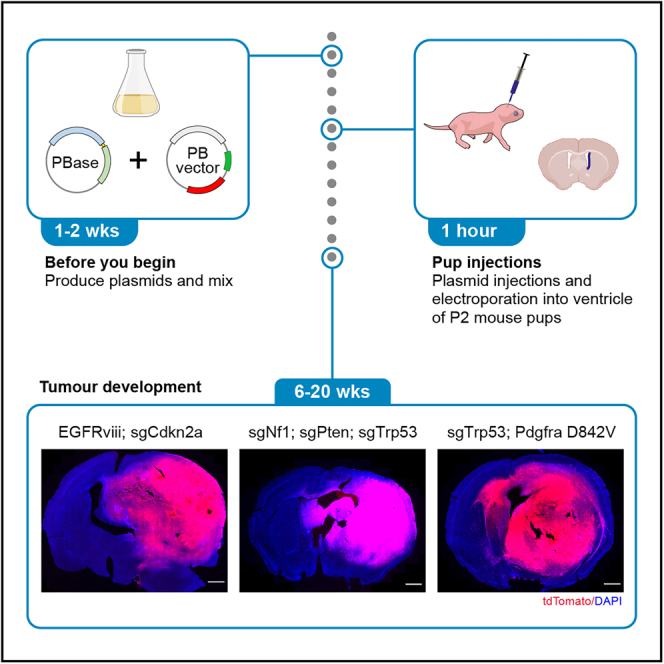

## Before you begin

**Figure 1:**
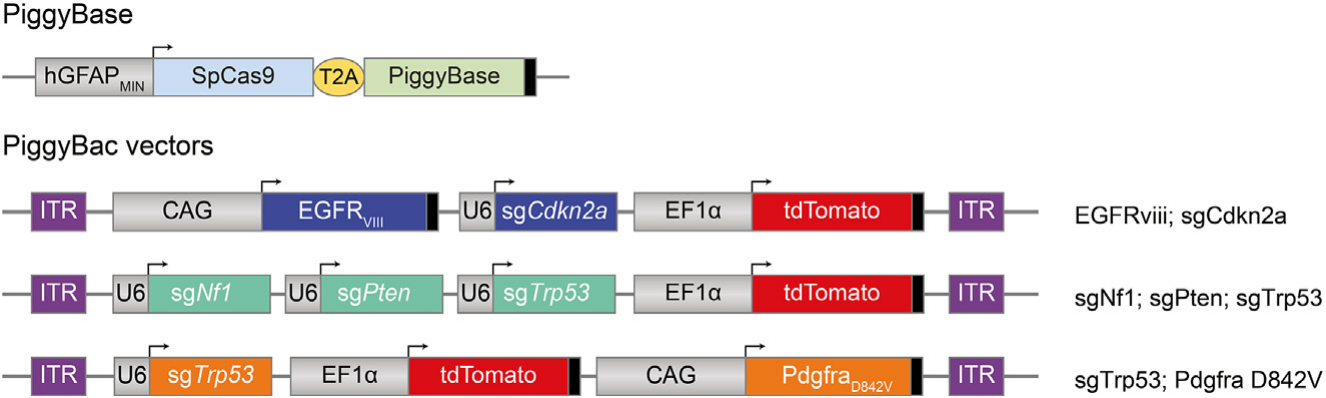
Plasmids for generation of glioblastomas in mice Schematic of constructs used to generate somatic glioblastomas in mice. For each GBM model, the piggyBase plasmid is mixed with one of the three piggyBac vectors. piggyBase and Cas9 expression is restricted to neural stem cells of the SVZ by hGFAPminimal (hGFAPmin) promoter. In piggyBac constructs, U6 promoter is used to drive expression of each CRISPR single guide RNA (sg). Overexpression of oncogenes is achieved with CAG promoter. Each piggyBac construct contains tdTomato reporter, to label all cells that have been targeted and their progeny. Black boxes indicate poly(A) signals.

This protocol describes the surgical procedure for co-electroporation of two plasmids targeting neural stem cells (NSCs) in the lateral ventricle of mouse postnatal day 2 (P2) pups: a nonintegrating plasmid encoding for the piggyBase transposase and Cas9 and an integrating piggyBac vector carrying the oncogenes, CRISPR guide RNAs and a tdTomato fluorescent reporter protein flanked by inverted terminal repeats (ITRs) (Figure 1). Upon electroporation, transient Cas9 expression results in inactivation of the tumor suppressor genes, whereas piggyBase-mediated integration of the piggyBac cargo ensures stable expression of the oncogenes and fluorescent reporters in the targeted NSCs and their progeny. Integration is mediated by the enzymatic activity of the piggyBase transposase which recognizes the ITRs and inserts them alongside their contents at TTAA sites within the recipient cell’s genome via a cut and paste mechanism. Targeting of NSCs is achieved by the minimal human GFAP (hGFAPmin) promoter sequence^1^^,^^2^^,^^3^ driving expression of the piggyBase/Cas9.

This protocol builds on previously developed methods for postnatal electroporation to deliver transgenes to subventricular zone (SVZ) cells *in vivo*, including for GBM development.^4^^,^^5^^,^^6^^,^^7^^,^^8^^,^^9^^,^^10^^,^^11^ Here by combining this technique with piggyBac transposition and CRISPR-Cas9 mediated gene editing, we developed ‘all in one’ constructs for generation of GBM in wild-type mice through delivery of key human driver mutations^12^^,^^13^ to endogenous NSCs. The system is safe, efficient and highly tractable, enabling delivery of any combination of driver mutations and targeting of other SVZ progenitor cells by using alternative promoters, e.g., NG2 for targeting oligodendrocyte progenitor cells.^14^ In addition, the large cargo size of the piggyBac system and the ease at which it can be further genetically engineered through simple cloning techniques or combined with existing transgenic or knock-out lines, makes this system suitable for virtually unlimited gain-and-loss of function or reporter studies.^1^^,^^15^ tdTomato enables identification of tumor cells within the mouse brain, and can be used for subsequent purification, if required.

### Institutional permissions

All animal procedures are performed in compliance with the Animal Scientific Procedures Act, 1986 and approved by the UCL Animal Welfare and Ethical Review Body (AWERB) in accordance with the international guidelines of the Home Office (UK).

Investigators utilizing this protocol must acquire the relevant permissions for working with animals from their institution and their relevant governing body.

### Preparation of injection capillaries

**Timing: 30 min**

You will need to pull capillaries for injection of pups before starting the procedure. Pulled capillaries can be stored in a dust-free environment (such as in a closed 15 cm tissue culture plate, secured to the base of the plate with blue tack) at 15°C–25°C indefinitely.

1. Pull borosilicate capillaries (1.0 mm O.D. × 0.58 mm I.D) using a Sutter capillary puller with the following settings:
  a. Heat = 98.
  b. Pull = 120.
  c. Velocity = 100.
  d. Time = 15.

***Note:*** Handle glass capillaries with care. Sharps should be disposed of according to local regulations.

***Note:*** Capillaries can also be pulled by hand over a flame if local regulations allow the use of naked flames.

### Preparation of plasmids

**Timing: 2 days to 2 weeks**

Before you begin with the surgeries, the plasmids which will be injected into pups need to be generated and prepared as an electroporation-ready mixture. The specific genetic composition of these plasmids is determined by the deletions or insertions that each project requires, and which cell type within the subventricular zone is to be targeted, as stated above.

***Optional:*** Modify constructs by InFusion cloning (https://www.takarabio.com/products/cloning/in-fusion-seamless-cloning) following plasmid design using SnapGene software. Original plasmids are detailed in Garcia Diaz et al.^1^ and modifications to allow tumor generation in wild-type mice are shown in Figure 1 and Table 1.

2. Transform plasmids into Stbl3 Chemically Competent *E. coli* cells.

**CRITICAL:** Plasmids contain highly repetitive regions; therefore, the use of an *E. coli* strain designed for cloning unstable inserts (such as Stbl3) is critical to reduce likelihood of recombination.

3. Perform maxiprep to obtain endotoxin-free stock of both piggyBase and piggyBac plasmids using a commercial kit, such as ZymoPURE II Plasmid Maxiprep Kit.

***Note:*** Store concentrated plasmids at ‒20°C.

4. Quantify plasmid concentration using NanoDrop (or similar).

***Note:*** Accurate quantification of plasmid concentration and dilution is essential as successful integration of the piggyBac requires exact molar ratio of piggyBase.

5. Dilute plasmid stocks to required concentration for 1:1 molar ratio in sterile DNase-free water (see Table 1).

***Note:*** Calculations to determine the concentrations of piggyBac and piggyBase to provide a 1:1 molar ratio and how to modulate these concentrations to change the ratios if required, are shown in Table 1.

6. Mix plasmid stocks and add fast green dye to final concentration of 0.05% according to Table 2. For one litter, 9 μL piggyBac + 9 μL piggyBase + 2 μL 0.5% fast green should be sufficient.

**Table 2:** Plasmid mix

Reagent	Stock concentration	Volume
piggyBase plasmid	1 μg/μL	9 μL
piggyBac plasmid	see Table 1	9 μL
Fast green dye	0.5%	2 μL

***Note:*** We recommend a 1:1 molar ratio of piggyBase to piggyBac with a final concentration of 1 μg/μL stock of piggyBase as a starting point for injections. In some instances, it may be beneficial to increase the ratio of piggyBase to piggyBac, particularly if the cargo in the piggyBac is large. We recommend a maximum concentration of piggyBase of 1 μg/μL as higher concentrations of plasmids leads to cell death post electroporation.

***Note:*** Fast green dye is used to allow visualization of plasmid mix during injection (see below).

***Note:*** Any unused plasmid mix can be stored at ‒20°C and used up to 3 months later.

***Note:*** Quantification of plasmid concentrations (conc.) for other plasmids can be determined using the following equation:

$$Conc.\;of\;piggyBac\;\left(\mu g/\mu l\right)=\frac{Conc.\;piggyBase\;\left(\mu g/\mu l\right)\times Insert\;size\;including\;ITRs\;\left(bp\right)}{piggyBase\;plasmid\;size\;\left(bp\right)}$$

**Table 1:** Example of plasmid sizes and concentrations

Plasmid name	Plasmid size (bp)	Insert size including ITRs (bp)	Concentration for usage at 1:1 ratio (μg/μL)
piggyBase hGFAPmin-SpCas9-T2A-PBase	9572	N/A	1
piggyBac CAG-EGFRviii_U6-sgCdkn2a_EF1a-tdTomato	11884	9362	0.978
piggyBac U6-sgNf1_U6-sgPten_U6-sgTrp53_EF1a-tdTomato	7923	5401	0.564
piggyBac U6-sgTrp53_EF1a-tdTomato_CAG-Pdgfra^D842V^	12293	9771	1.021

## Key resources table

**Table:** 

REAGENT or RESOURCE	SOURCE	IDENTIFIER
**Bacterial and virus strains**		

One Shot Stbl3 chemically competent *E. coli* (alternatives available)	Thermo Fisher Scientific	Cat#C737303

**Chemicals, peptides, and recombinant proteins**		

Fast green (alternatives available)	Sigma	Cat#F7252
DNase-free water (alternatives available)	Invitrogen	Cat#AM9937
Dulbecco’s phosphate-buffered saline (alternatives available)	Sigma	D8537
IsoFlo	Apiece	5965901

**Critical commercial assays**		

ZymoPURE II Plasmid Maxiprep Kit (alternatives available)	Zymo Research	Cat#D4203
InFusion cloning kit (alternatives available)	Clontech	Cat#638917

**Experimental models: Organisms/strains**		

Mouse: C57BL/6J (alternatives available)	Charles River	RRID:IMSR_JAX:000664
Mouse: FVB/N (alternatives available)	Charles River	RRID:MGI:2165215

**Recombinant DNA**		

Plasmid: piggyBase hGFAPmin-SpCas9-T2A-PBase	Garcia-Diaz et al.^1^	N/A
Plasmid: piggyBac CAG-EGFRviii_U6-sgCdkn2a_EF1a-tdTomato	Garcia-Diaz et al.^1^	N/A
Plasmid: piggyBac U6-sgNf1_U6-sgPten_U6-sgTrp53_EF1a-tdTomato	Garcia-Diaz et al.^1^	N/A
Plasmid: piggyBac U6-sgTrp53_EF1a-tdTomato_CAG-PdgfraD842V	Garcia-Diaz et al.^1^	N/A

**Software and algorithms**		

SnapGene	N/A	RRID:SCR_015052

**Other**		

NanoDrop (alternatives available)	Thermo Fisher Scientific	Cat# ND-ONE-W
Micropipette puller (e.g., Sutter Instrument Model P-97) (alternatives available)	Sutter Instrument	Model#P-97
Borosilicate glass capillaries (1.0 mm OD × 0.58 mm ID)	Harvard Apparatus	Cat#30-0019
15 cm dishes (alternatives available)	Starlab	Cat#CC7672-3614
Microloader pipette tips	Eppendorf	Cat#5242956003
0.22 μm PVDF sterile syringe filter (alternatives available)	Starlab	Cat#E4780-1221
35 mm dishes (alternatives available)	Corning	Cat#430165
Isofluorane anaesthetic delivery system – small animal system	VetTech Solutions	Cat#AN071
2 mL syringe (alternatives available)	Appleton Woods	Cat#BD574
Lamp with swan arm (alternatives available)	Intralux	Cat#4000-1
FemtoJet 4i microinjector	Eppendorf	Cat# 5252000021
Electrode gel (alternatives available)	Compex	Cat#602047
BTX Gemini X2 electroporator	BTX	Cat#45-2006
Tweezertrodes attachment for electroporator	BTX	Cat# 45-0488
Surgical skin marking pen (alternatives available)	Schuco	Cat#Z-SS-665

## Materials and equipment

• 0.5% fast green: add 50 mg fast green powder to 10 mL of DNase-free H_2_O. Sterile filter using 0.22 μm PVDF sterile syringe filter.

Store at 15°C–25°C for up to a year unless precipitation occurs.

### Equipment setup details

• Micropipette puller (e.g., Sutter Instrument Model P-97).
  ○ For the Sutter Instrument above, we use the following settings:
    - Heat = 98.
    - Pull = 120.
    - Velocity = 100.
    - Time = 150.

***Alternatives:*** Glass capillaries can be pulled by hand over a flame.

• Eppendorf FemtoJet 4i Microinjector (Cat# 5252000021).
  ○ We recommend the following settings as a starting point:
    - pi 90 hPa. This setting may need to be adjusted depending on capillary bore size to provide optimal output flow from capillary.
    - pc 15 hPa.
• BTX Gemini X2 Electroporator (Cat# 45-2006) and Tweezertrodes Gap width: 7 mm (Cat# 45-0488).
  ○ We use the following settings:
    - Voltage: 100 V.
    - Duration: 50 ms.
    - Droop: 0.3%.
    - Number of pulses: 5.
    - Pulse Interval: 0.8 s.

## Step-by-step method details

### Setting up the equipment for pup injections

**Timing: 30 min**

**Figure 2:**
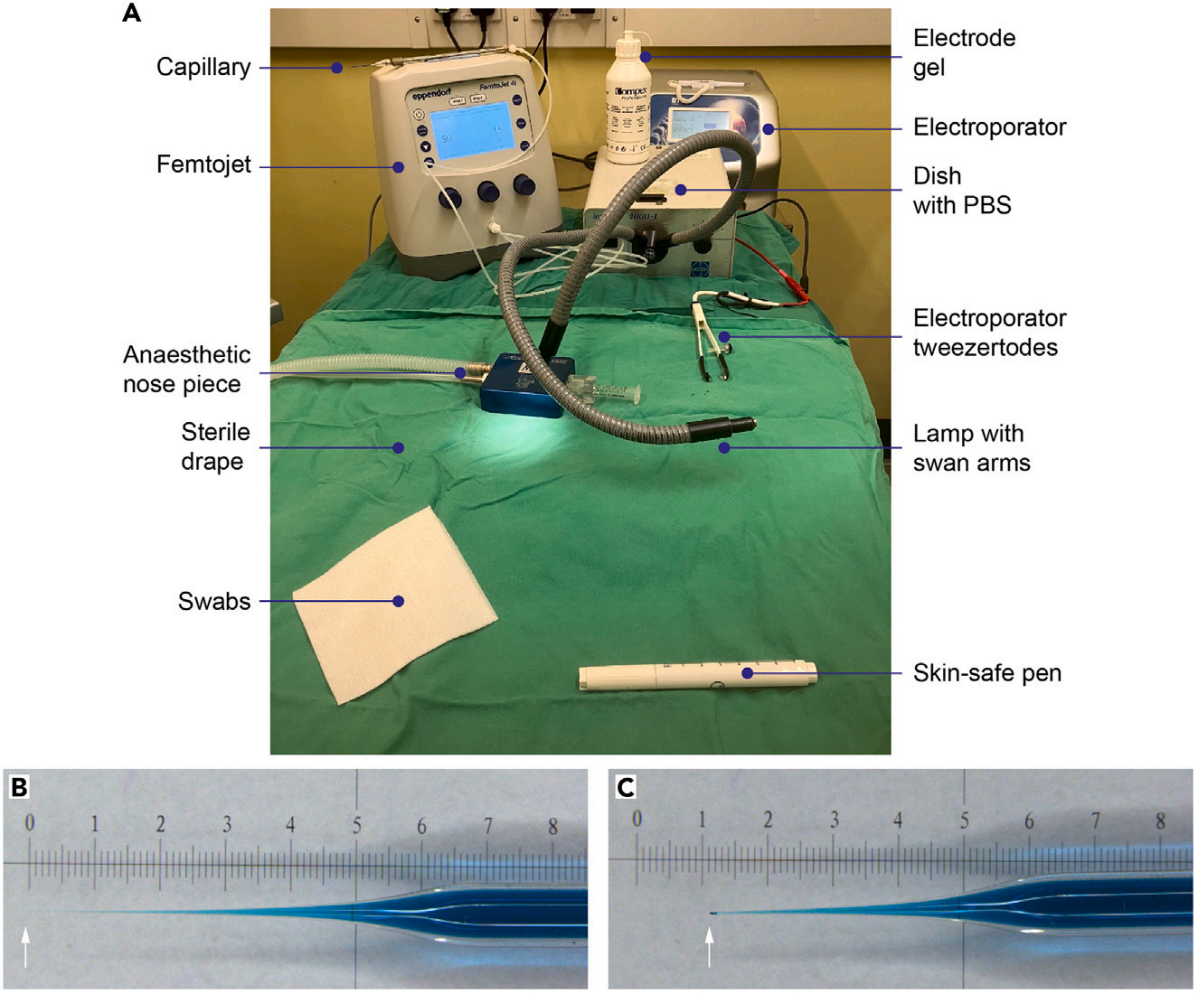
Preparation for pup injections (A) Set up of surgical area, with key equipment and materials labeled (Steps 1–6). (B and C) Capillary before (B) and after (C) the tip is broken (Steps 10–11).

This step describes the setup of the equipment required for intraventricular injection and electroporation of plasmids into the subventricular zone of mouse pups (Figure 2A). To minimize the time that pups are removed from their home cage it is important that all the equipment is ready prior to injections.

1. On a sterile surgical table set up the equipment required for the injections ensuring that all equipment is clean and sterile (Figure 2A).

**CRITICAL:** To prevent rejection of the pups by the parents following injection, no antiseptic skin cleansers are used during this procedure. Thus, the procedure cannot be maintained in totally aseptic conditions. However, to minimize any chance of post-surgical infections all equipment should be sterile before starting, and sterile gauze used where necessary.

2. Place a heat mat or warming plate set to 37°C under a sterile drape.
3. Set up the FemtoJet with foot paddle attached.
  **CRITICAL:** Do not attach the capillary while the FemtoJet is pressurizing.
  ***Note:*** Once the FemtoJet is pressurized the pumping noise will stop and the FemtoJet will be ready for attachment of the capillary needle holder.
  a. Press the menu button and select “capillary may be changed” before attaching the capillary cable and needle holder to the machine.
  b. Press the menu button again to return to the injection screen.
    **CRITICAL:** The FemtoJet must be in “capillary may be changed” mode when a new capillary is attached to maintain the pressure of the FemtoJet.
4. Place a nosepiece attached to an isoflurane anesthetic delivery system on the heat pad.

***Note:*** If the nosepiece is too large for a pup, then attach a 2 mL syringe to provide a suitable head holder for the pup to enable efficient delivery of the anesthesia.

5. Place a suitable light source on the table, so light can be directed through the head of the pup, illuminating the ventricles in the brain.
6. Set up the square wave electroporator (BTX Gemini X2 Electroporator).
  a. Attach platinum tweezertrodes (7 mm) and foot pedal.
  b. Program the following settings:
    i. Voltage 100 V.
    ii. Duration 50 ms.
    iii. Droop 0.3%.
    iv. Number of pulses 5.
    v. Pulse interval 0.8 s.
7. Coat the electroporator tweezertrode plates with electrode gel.
  a. Prime the electroporator so it is ready for pulse delivery.

***Note:*** If the tweezertrodes cables are not correctly inserted into the electroporator, an “Interlock Open” error message will appear and the electroporator will not deliver electric pulses. In such case, make sure all connections are tight and reprime the electroporator.

8. Fill a 35 mm dish with PBS and place it within reach of the capillary.

***Note:*** This will be used to check that plasmid is being correctly expelled from the capillary (preparation of the capillary will be described in the next section: filling the capillary).

### Filling the capillary

**Timing: 5 min**

This step describes the process of filling the capillary and creating an injection needle that is the optimal size for delivery of plasmids to the ventricle (see Figures 2B and 2C and Methods video S1).

9. Using an extended pipette tip attached to a 20 μL pipette, pipette 10 μL of plasmid mix slowly into the extended pipette tip (see Methods video S1).
10. Fill the capillary from the tip upwards with the entire contents of the pipette, slowly extracting the pipette tip whilst continuing to pipette the plasmid.

***Note:*** Avoid creating bubbles as the capillary is filled.

**CRITICAL:** Pipette tips must be fine and long enough to reach the end of the pulled capillary. We recommend Eppendorf microloader 20 μL tip.

***Note:*** Handle glass capillaries with care to avoid needlestick injury. Sharps should be disposed of according to local regulations.

11. Break the tip of the capillary gently against a solid object, such as a 35 mm dish, as shown in Figures 2B and 2C and Methods video S1.
12. Attach the filled capillary to the FemtoJet needle holder (still under “capillary may be changed” setting).
  a. Cancel capillary change setting to enable injection.
  b. Test that the bore of the capillary is correct by depressing the FemtoJet foot pedal with the capillary tip dipped in PBS.

***Note:*** Fast green colored plasmid mix should be expelled from the capillary into the PBS in a steady stream. If the plasmid is not being released it is likely that the bore of the capillary is too small. Change the FemtoJet setting to “capillary may be changed” and repeat Steps 11–12.

***Note:*** If the capillary still does not expel plasmid after you have re-broken the tip there may be a blockage in the capillary holder. If so, remove the capillary (“capillary may be changed” setting) and press the “clean” button on the right-hand side of the FemtoJet. This expels air through the needle holder and will clear any blockage. Repeat Step 12.

Methods video S1. Filling of pipette and preparation of capillary for injection, related to steps 9–11

### Intraventricular pup injections and electroporation

**Timing: 30 min**

This step provides a detailed description of the injection protocol including handling of the pups and minimizing the disturbance to the parental mice so injected pups are welcomed back into the litter. The best results are obtained when the pups are removed from the cage for the least amount of time. Pups from any mouse strain can be used, including genetically modified strains, according to experimental requirements. Here we have used C57BL/6J or FVB/N mice. See also Methods video S2.

**Figure 3:**
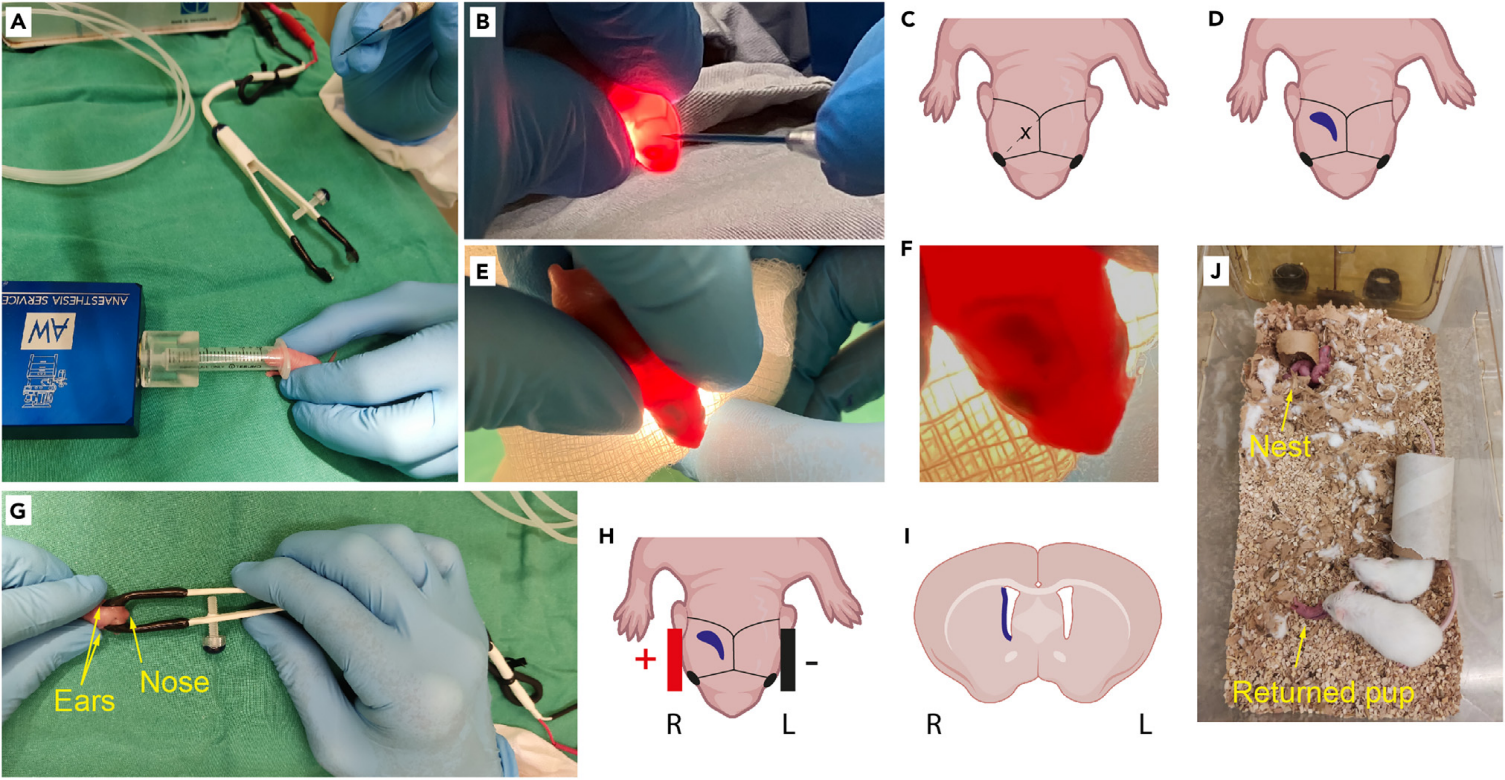
Surgical procedure (A) Pup is anaesthetized. Operator holds pup in one hand and capillary in other. (Step 13). (B and C) Pup is injected. Graphical representation in C shows area of injection marked by X. (Step 15–16). (D‒F) Successful injection shows crescent of filled ventricle visualized using fast green. (Step 16). (G and H) Electroporation. (Steps 17–18). (I) Schematic coronal section indicating the expected location of targeted cells along the SVZ after successful injection and electroporation. (J) Pups are returned to home cage away from the nest. Parent will collect and return the pup to the nest. (Step 21). See also Methods video S1.

***Note:*** Hold the pup in one hand and have the capillary in the other hand ready for injection to maximize the time the pups remain immobilized and minimize the time pups are out of their home cage (Methods video S2).

13. Remove P2 pup from the nest taking care not to disturb the other pups.
  a. Place the pup in the syringe under the isoflurane stream (3%) and hold in place until the breathing rate slows and the pup becomes immobile (Figure 3A).

14. Remove the pup from the isoflurane stream and place over a light source so the light penetrates through the brain showing the position of the ventricle (Figure 3B and Methods video S2).

**CRITICAL:** Successful injection and subsequent development of tumors requires an accurate assessment of pup age. We have optimized our GBM models for injections at P2, but injections at P1 or P3 is also possible with this technique. However, injection at P1 may lead to shorter tumor latency and resulting tumors have not been as extensively characterized. It is more difficult to visualize the ventricle of P3 pups for accurate injection, as their skin is more pigmented. It is therefore essential that the litters are identified as soon as they are born as consistency of injection timing will improve outcomes.

15. Locate the ventricle and insert the capillary directly into the ventricle on the right side of the head, at the halfway point on an imaginary line between the eye and lambda suture (Figures 3B and 3C).
16. Depress the foot pedal and inject the plasmid into the ventricle until the entire ventricular cavity is filled.
  ***Note:*** Complete filling of the ventricle is achieved when the fast green dye reveals the classic crescent shape of the ventricle (Methods video S2, Figures 3D‒3F). To fill the whole ventricle, you may need to inject more than once by depressing the foot pedal.
  a. Once the ventricle is full, remove the capillary.
  b. Move the pup from the light source and hold gently on the surgery table for the electroporation process (Figure 3G).
    **CRITICAL:** If the capillary is not in the correct position, the ventricle will not fill with plasmid. In case of unsuccessful injection, it is not advisable to attempt re-injection as non-SVZ GFAP positive astrocytes may be targeted due to residual misplaced plasmid from the first injection. Animals incorrectly injected should be sacrificed in accordance with local regulations.
    ***Note:*** The volume of plasmid mix injected with each depression of the foot pedal is dependent on the bore of the capillary, and the length of time the foot pedal is depressed. Injection is continued until the ventricle is full, which corresponds to approximately 1 μL of plasmid mix per pup.
    **CRITICAL:** Do not overfill the ventricle. If you continue to inject once the ventricle is full, plasmid mix will overflow from the injection site. Animals incorrectly injected should be sacrificed in accordance with local regulations.
17. Place the gel-covered tweezertrodes either side of the head just in front of the ears, with the positive pole adjacent to the injected ventricle.

***Note:*** DNA is negatively charged so it will be attracted to the positive electrode (Figures 3G‒3I).

18. Press the electroporator foot pedal **once** to activate the electroporator and deliver the electroporation pulses (5 pulses will be delivered).

**CRITICAL:** The pulses delivered from the electroporator create a mild shock through the pups so ensure that the tweezertrodes do not move during this process.

***Note:*** If no current is delivered, check for error messages on the electroporator and that the foot pedal is correctly attached. If not, make sure all connections are tight and reprime the electroporator (Step 7).

**CRITICAL:** Do not delay between the end of the injection process and activation of the electroporator as the plasmid will leak through the brain if there is a long delay before electroporation.

19. Clean the electrode gel from the head of the pup using a sterile gauze.

***Note:*** Electrode gel remaining on the pups may lead the mum to reject the pups when they are returned to the cage so make sure the pups are as clean as possible.

20. Using a surgical skin safe marking pen, mark the back of the pup so injected pups can be recognized from their non-injected littermates.
21. Return the pup to parental cage, placing the pup away from the nest.
  a. Observe while the adult mice collect the pup and return it to the nest (Figure 3J).

***Note:*** Some mice take a while to retrieve their pups and return them to the nest. Try not to interfere with the mice while the pups are being injected. If none of the pups have been returned to the nest once all pups are injected, then move the pups into the nest and check the mother is nursing the pups after 30 minutes.

22. Repeat steps 13 to 21 with the rest of the litter.

**CRITICAL:** Before proceeding to step 13, ensure that the capillary contains a volume of plasmid mix in excess of that required for the next injection (greater than 1 μL). This prevents injection of air into the ventricle, which may lead to subsequent complications, such as hydrocephalus.

Methods video S2. Pup injection and electroporation procedure, related to steps 13–21

## Expected outcomes

**Figure 4:**
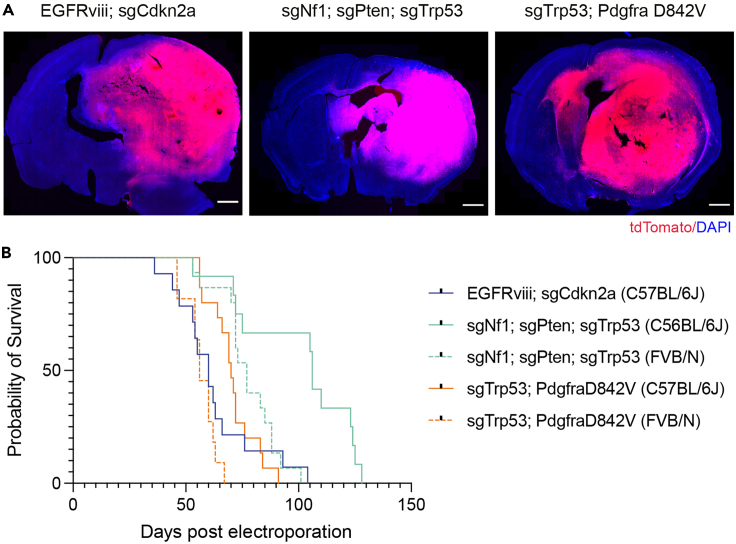
Expected outcomes (A) Representative images of coronal sections from tumors generated with this protocol. Tumor cells express tdTomato fluorescent protein. Nuclei are labeled with DAPI. Scale bar = 1 mm. (B) Kaplan-Meier survival curves for mice bearing tumors produced with this protocol. Kaplan-Meier curves with dotted lines indicate reduced latency in mice from an FVB/N background compared to C57BL/6J mice with the same mutations. EGFRviii; sgCdkn2a (C57BL/6J): n = 14, Median survival = 60d (days post electroporation). sgNf1; sgPten; sgTrp53 (C56BL/6J): n = 12, Median survival = 106d. sgNf1; sgPten; sgTrp53 (FVB/N): n = 15, Median survival = 77d. sgTrp53; PdgfraD842V (C57BL/6J): n = 15, Median survival = 70d. sgTrp53; PdgfraD842V (FVB/N): n = 11, Median survival = 56d.

Following successful injection and electroporation, all pups are expected to survive the procedure and go on to develop tumors at >98% penetrance (Figure 4).^1^^,^^15^

The latency of tumor development will depend on the mutations that have been introduced and the genetic background of the recipient mice (typically 6–20 weeks, see Figure 4). Differences in resulting tumor location and size are common within and between litters as observed in genetically engineered mouse models (GEMMs).^14^ For survival analysis, at least 10 mice per experimental paradigm are advisable, depending on expected effect size and power calculations. If tumors are to be used for immunohistochemical/flow cytometry analysis, we would recommend a minimum of 4 animals per group.

To check that injections and plasmids are working as expected, we suggest you collect some of the mice at early time points (e.g., 3 weeks post-injection) to check that your cells of interest have been targeted correctly and genes of interest overexpressed or deleted. If a fluorescent reporter is included in the construct, successfully targeted cells and their progeny will express this protein. Successful integration of plasmids can also be determined by preparing neural stem cells from injected pups at P10-P14^16^ and carrying out Western blots or immunohistochemistry to identify overexpressed or deleted genes.

## Limitations

Efficiency of tumor development depends on the success of the injections and technical skill of the user. If targeting is inaccurate, tumors will not form. In cases of successful injection, we have observed a penetrance of >98%.

Although the piggyBac system tolerates a large cargo size, we have found that larger inserts (>12 kb) may compromise integration efficiency and target cell viability. Consideration should be taken when designing further modifications to the constructs.

In this system, transformation results from stochastic integration of the constructs into endogenous neural stem cells. It is therefore not possible to precisely control the number of cells that are targeted, leading to variability in the latency of subsequent tumor development comparable to, or lesser than, what is commonly observed in GEMMs of GBM.^14^

This approach targets many neural stem cells per mouse; therefore, the resulting tumors are, by nature, polyclonal. Furthermore, random integration of the piggyBac may result in different clones having variable levels of transgene expression.

## Troubleshooting

### Problem 1

**Figure 5:**
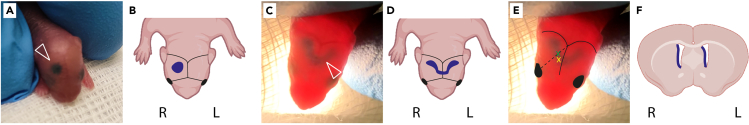
Potential problems that may be encountered (A and B) Unsuccessful injection leads to plasmid mix pooling under the surface of the skin with no visible crescent-shaped filling of the ventricle (white arrowhead). This is likely caused by the injection being too superficial. The plasmid mix will pool in the cortex or above the skull. Pup should be humanely culled. (C‒F) Injection leads to filling of both ventricles (white arrowhead). Representation in E of incorrect capillary entry site used for this injection (yellow cross). Correct location for injection is indicated (green cross). F Coronal section representation of the locations of targeted cells from injection C–E.

No fast green is seen in the ventricle following injection but there is fast green visible under the skin (related to Step 16, see Figures 5A and 5B).

### Potential solution

• Injection was not in the correct place. Make sure that the capillary is in the correct place (Figures 3B and 3C) and the crescent fills as the injector pedal is depressed.
• If the injection is in the correct place but no plasmid (no visible fast green) is entering the ventricle, the capillary may be blocked. Remove the capillary and check delivery by ejecting some plasmid into the PBS dish. If there is no plasmid being expelled from the capillary, re-break the tip (with FemtoJet set on “capillary may be changed”) and check again for delivery of the plasmid into PBS. If the capillary is working correctly, you can re-start the pup injection process.

### Problem 2

Both ventricles are filled with plasmid as indicated by fast green appearance in both hemispheres (related to Step 16, Figures 5C–5F).

### Potential solution

It is likely that the injection has been successful, but the position of the capillary was slightly too anterior and the plasmid has crossed the midline into the opposite ventricle (see Figure 5E). Electroporation will then lead to targeting of the cells in the lateral wall of the sub-ventricular zone in the right hemisphere, but also in the septal wall of the left ventricle (Figure 5F). For some experiments this may be a desirable outcome, but if not then adjust capillary placement for further mice in the litter.

### Problem 3

No fluorescent cells visible following injection (related to expected outcomes).

### Potential solution

• The plasmid mix was not correctly prepared. Check whether any cells were targeted in any of the litter. If not, then prepare a new plasmid mix and inject a new litter.
• The ratio of piggyBase:piggyBac may not be optimal for integration of the cargo. Try altering the ratio of piggyBase to piggyBac, particularly if you have a large cargo within the piggyBac plasmid. We suggest increasing the ratio to 2:1 if the cargo is large.
• If the injection is in the correct place, and the piggyBase:piggyBac ratio is optimal, but there are still no targeted cells following injection, the cargo in the piggyBac maybe toxic to the cells. Test transfection in neural stem cells *in vitro* to assess if they survive integration of the plasmid and express the fluorescent reporter or collect brain tissue within 2–3 days following electroporation.
• The concentration of the piggyBase:piggyBac plasmid mix may be too concentrated which could lead to cell death. Reduce the concentration of your mix to below 2 μg/μL.

### Problem 4

Fluorescent cells were identified in the lateral septal nucleus but not in the subventricular zone (related to expected outcomes).

### Potential solution

• The tweezertrode paddles were the wrong way round thus targeting the lateral septal nucleus rather than the lateral ventricle. Switch the paddles around so that the positive electrode (indicated by the plastic screw Figure 2) is adjacent to the side of the head that was injected.

### Problem 5

Following injection, cells express the fluorescent protein, but no tumors develop subsequently (related to expected outcomes).

### Potential solution

• If you have targeted the correct cells but no tumors have developed, it is possible that the genes of interest may have not been deleted or overexpressed. Test the efficiency of the constructs *in vitro* by preparing primary NSC cultures from the electroporated SVZs and assessing expression levels of the targeted genes using standard molecular biology techniques.^15^

### Problem 6

No pups remain the day after injection (related to Step 21).

### Potential solution

• There is a possibility that the parents have cannibalized the pups. Before injection check that there are milk spots in the pups and that they are being fed by the mother.
• When returning the pups to the cage, make sure that there is no electrode gel on the pups’ head which may lead to the parents not reintroducing the pups to the nest.
• Try and minimize the time taken for the injection and electroporation process to ensure that the pups are away from the litter for the shortest possible time, so they are not rejected and then eaten.

## Resource availability

### Lead contact

Further information and requests for resources and reagents should be directed to and will be fulfilled by the lead contact, Simona Parrinello (s.parrinello@ucl.ac.uk).

### Technical contact

Questions about the technical specifics of performing the protocol should be directed to the technical contact, Melanie Clements (m.clements@ucl.ac.uk).

### Materials availability

There are restrictions to the availability of plasmids due to requirement for MTA. Please contact the lead contact for requests as above.

### Data and code availability

This study did not generate any datasets or code.
